# Multiple Attention Mechanism Enhanced YOLOX for Remote Sensing Object Detection

**DOI:** 10.3390/s23031261

**Published:** 2023-01-22

**Authors:** Chao Shen, Caiwen Ma, Wei Gao

**Affiliations:** 1 Xi’an Institute of Optics and Precision Mechanics, Chinese Academy of Sciences, Xi’an 710119, China; 2 University of Chinese Academy of Sciences, Beijing 100049, China

**Keywords:** remote sensing, object detection, multiple attention, CBAM, Swin Transformer, loss function

## Abstract

The object detection technologies of remote sensing are widely used in various fields, such as environmental monitoring, geological disaster investigation, urban planning, and military defense. However, the detection algorithms lack the robustness to detect tiny objects against complex backgrounds. In this paper, we propose a Multiple Attention Mechanism Enhanced YOLOX (MAME-YOLOX) algorithm to address the above problem. Firstly, the CBAM attention mechanism is introduced into the backbone of the YOLOX, so that the detection network can focus on the saliency information. Secondly, to identify the high-level semantic information and enhance the perception of local geometric feature information, the Swin Transformer is integrated into the YOLOX’s neck module. Finally, instead of GIOU loss, CIoU loss is adopted to measure the bounding box regression loss, which can prevent the GIoU from degenerating into IoU. The experimental results of three publicly available remote sensing datasets, namely, AIBD, HRRSD, and DIOR, show that the algorithm proposed possesses better performance, both in relation to quantitative and qualitative aspects.

## 1. Introduction

Object detection aims to find the position and size of all relevant objects in the image. Remote sensing images contain sensitive objects, such as vehicles, aircraft, buildings, and forests. Recently, remote sensing images have been developed towards higher spatial, spectral, and temporal resolutions, which greatly enriches the information of remote sensing images. How to use this remote sensing information more effectively has become a core issue in technology applications.

Remote sensing object detection is an essential application of remote sensing [1] information. It is widely used in various fields, such as land use and cover statistics, environmental monitoring, geological disaster investigation, Geographic Information System updates, precision agriculture urban planning, military defense, etc. In recent years, technologies, such as the watershed segmentation algorithm [2], visual saliency algorithm [3], canny edge detection algorithm [4], and classification algorithm based on SVM [5], have been applied to the object detection of RGB images and have achieved good results against simple backgrounds. However, in optical remote sensing imaging, the shooting angle is not horizontal, and most captured objects are tiny. The remote sensing imaging process is also easily affected by complex background factors, such as light intensity, shooting time, and weather. As a result, detection accuracy and speed become worse when the above object detection methods are applied to remote sensing images. With the widespread use of deep learning methods in object detection, the accuracy of object detection has been dramatically improved. Currently, object detection techniques are mainly divided into two categories.

The first category is two-stage algorithms, mainly represented by the R-CNN series, mainly including R-CNN [6], Fast R-CNN [7], and Faster R-CNN [8]. The above two-stage object detection algorithms have high accuracy, but run slow. Lin Na et al. [9] used the idea of hollow residual convolution to extract shallow features. They fused the shallow information with deep features, which effectively improved the detection accuracy of aircraft in remote sensing images. Yao Yanqing et al. [10] used a dual-scale feature fusion module to alleviate the loss of deep information and effectively improve the detection ability of multi-scale remote sensing objects. Zhang Xiaoya et al. [11] proposed a remote sensing-based object detection algorithm with a multi-stage cascade architecture, enhancing both the detection tasks of the horizontal frame and the rotating frame. According to the characteristics of different types of aircraft, such as the size scale not being fixed, Dong Yongfeng et al. [12] proposed an object detection method based on Mask RCNN. Dai Yuan et al. [13] proposed remote sensing image object detection based on an improved rotation region generation network. This method was improved based on Faster R-CNN. The test results in the DOTA dataset show that this algorithm improves detection accuracy. The above methods all adopt a two-stage convolution neural network algorithm and have good accuracy; however, the detection efficiency is low, and it is not suitable for mobile terminals.

The second category is single-stage algorithms, represented by the YOLO series, such as YOLO [14], YOLOv2 [15], YOLOv3 [16], YOLOv4 [17], and YOLOX [18]. These single-stage detection algorithms only use one convolutional neural network to locate and directly classify all objects, reducing the step of generating region proposals. Based on YOLOv3, Zhang Yu et al. [19] proposed a multi-scale feature densely connected remote sensing object detection model, YOLO-RS, which retained more feature information in the image and improved the information interaction between feature layers of different scales. Zhang Tianjun et al. [20] proposed improving the remote sensing image aircraft object detection method based on YOLOv4. This method greatly improves the detection accuracy of UCAS-AOD and RSOD public remote sensing datasets, which is conducive to the rapid detection of remote sensing image aircraft objects in actual industrial scenes. Lang Lei et al. [21] proposed a lightweight remote sensing image object detection model based on YOLOX Tiny based on YOLOX, evaluating the effectiveness of the algorithm on the public remote sensing image object detection dataset DIOR, and the test results proved the method’s improved detection accuracy. In addition, compared with two-stage object detection algorithms, the detection speed of one-stage algorithms is greatly improved.

The above object detection algorithms are all improvements based on convolutional neural networks. Although convolutional neural networks can effectively extract local information, their ability to obtain global context information is limited. Based on the self-attention mechanism, Transformer [22] retains enough spatial information for object detection through the multi-head self-attention module, which is beneficial to improve the detection performance of tiny objects. Compared with CNN, Transformer obtains global features from shallow layers and contains more spatial information. Transformer has superior performance in parallel computing in the current hardware environment (GPU). However, when used on remote sensing images with large-scale changes and uneven distribution of object sizes, Transformer faces a large amount of calculation, and the real-time performance is poor. In 2021, Liu et al. proposed the Swin Transformer architecture [23], built by replacing the standard multi-head self-attention (MSA) module in the Transformer block with a shifted window-based module, while keeping other layers unchanged. Swin Transformer is hierarchically produced to solve multi-scale problems and provide dimensional information on each scale. Swin Transformer uses shifted windows to allow interaction between adjacent windows, which expands the receptive field, improves efficiency, and reduces computational complexity. Based on Swin Transformer, Xu et al. [24] proposed a Local Perception Swin Transformer (LPSW) to explore remote sensing object detection and instance segmentation to enhance the network’s local perception ability and improve the detection accuracy of small-scale objects.

Although there have been a great number of studies regarding object detection for remote sensing images, the detection algorithms still lack the robustness to detect tiny objects against complex backgrounds. 

Therefore, in this paper we propose a Multiple Attention Mechanism Enhanced YOLOX [25] (MAME-YOLOX) algorithm to address the above problem. The main contributions are threefold:

(1) We introduce the CBAM [26] attention mechanism into the backbone of the YOLOX, so that the detection network can focus on the saliency information.
(2) Based on the idea of the self-attention mechanism, the Swin Transformer is also integrated in the YOLOX’s neck module. This module is used to identify the high-level semantic information and enhance the perception of local geometric feature information.
(3) Instead of GIOU loss, CIoU loss [27] is adopted to measure the bounding box regression loss, which can prevent the GIoU from degenerating into IoU. This design can improve the accuracy of the predicted bounding box.

The remaining sections of this paper are organized as follows. The related works are reviewed in Section 2. The proposed method is described in Section 3. Section 4 shows the experiment and analysis. Finally, we present the conclusion in Section 5.

## 2. Related Works

### 2.1. YOLOX

YOLOX [28] consists of four parts: input, a backbone for feature extraction, a neck for feature fusion, and prediction. 

The input part includes Mosaic Augmentation, Mixup Augmentation, and Focus architecture. Mosaic Augmentation reads four pictures at a time, performs operations such as scaling and flipping on the four pictures and combines them into one image. Through Mosaic Augmentation, the background of the dataset is enriched, the training speed of the network is improved, the uneven distribution of objects is changed, the system’s robustness is improved, and the consumption of GPU memory is reduced. MixUp is an improved strategy based on Mosaic. MixUp fuses two images according to a specific fusion coefficient, achieving the same function as Mosaic. Focus extracts a value for every other image pixel, slices to obtain independent feature layers, then stacks to multiply channels by a factor of 4.

The backbone is the feature extraction network and also the main body of YOLOX. The backbone of YOLOX is CSPDarknet, which consists of the residual block, CSP block, and SiLU. The residual network effectively alleviates the gradient disappearance problem in deep neural networks. CSPBlock dramatically improves the computing and learning ability of CNN and reduces the amount of calculation. The activation function, SiLU, is an enhanced version of Sigmoid and ReLU. It is smooth, unbounded, and non-monotonic and performs better than ReLU in deep networks.

The neck is used for feature fusion, and its core is the feature pyramid network (FPN) and path aggregation network (PAN) [29,30]. With the deepening of the convolutional layer, the features of large objects in the high layer are rich, while in the low layer, the location information of large objects and the category features of tiny objects are better. The workflow is as follows. First, FPN is used to transfer and fuse the high-level feature information by up-sampling, and then the predicted feature map is obtained by down-sampling and fusion through PAN. FPN improves the detection ability of tiny objects, and PAN better transmits the bottom layer information to the top layer.

Prediction is a classification and regression for YOLOX. Three feature layers of different scales are obtained through the neck and then, respectively, used to identify large, medium, and small objects. Each feature layer can be regarded as a collection of feature points, and each feature point has a position parameter and the number of channels. Prediction finds whether there is an object corresponding to the feature point. Prediction comprises Decoupled Head, Anchor Free, SimOTA, and LOSS. Decoupled Head con-verges faster and with higher precision, but the computational complexity also increases. Compared with other anchor-based methods, Anchor Free detectors have two-thirds fewer parameters, run faster, and perform better. SimOTA can perceive loss and quality, provide a center point prior, dynamically change the number of positive anchors, and cover the global view. As a result, it is used as an advanced label assignment mechanism.

The head of YOLOX is different from the previous YOLO head. The decoupling classification and regression of the previous version are implemented in one convolution, while the decoupling of the YOLO head in YOLOX is implemented in two parts.

The SimOTA screens the feature points to obtain the candidate bounding boxes of positive samples. Firstly, it defines a loss function representing the relationship between the ground truth and the predicted boxes. Then, it filters out the anchors with the center falling within the range of the ground truth or a square box, of which the center is the same as the ground truth. The anchors picked out are called candidates. Then, it calculates the intersection over union (IOU) [31] between the ground truth and its candidates. Then, it adds up the top ten largest IOUs as the k value of this ground truth, which means that k feature points correspond to the ground truth. Finally, it calculates the classification accuracy of every ground truth and its candidates, by which it obtains the cost function. The k points with the lowest cost are the positive samples of the ground truth. 

In YOLOX, the bounding box regression loss function is GIOU loss. The definition of GIOU loss is shown in Equation (1).

(1) $$GIOU=IOU-\frac{\left|C-(A\cup B)\right|}{\left|C\right|}$$

The classification loss of the YOLOX is cross entropy (CE), while the GIOU loss is used to measure the intersecting scale between the predicted bounding box and the ground truth. Assuming C is the smallest rectangular frame containing A and B, when IOU = 0, the distance between A and B is great, and GIOU tends to -1. This solves the problem of the loss function not being derivable when the bounding boxes do not coincide and the IOU loss does not correctly reflect the intersection situation when the size of the two predicted boxes is the same, which means that the IOU is the same too. However, if multiple prediction boxes of the same size are inside the ground truth, each minimum box C is the same, and the union of A and B is also the same. At this time, GIOU loss cannot correctly reflect the prediction boxes’ intersection situation.

### 2.2. CBAM

**Figure 1:**
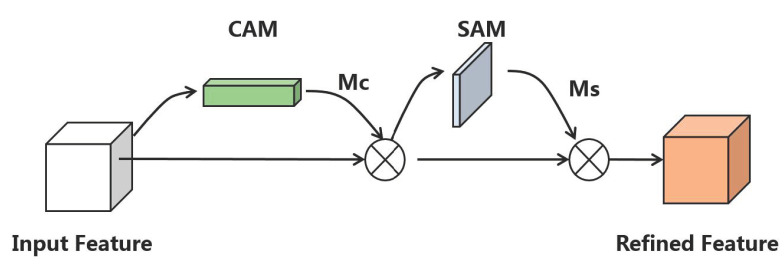
The architecture of the CBAM.

The CBAM [32] uses the channel attention module (CAM) and the spatial attention module (SAM) to learn where and what to focus on so that it pays more attention to essential features while ignoring unnecessary ones. The architecture of the CBAM is shown in Figure 1.

The CBAM workflow is as follows. Firstly, given a feature map $F\in R^{H\times C\times W}$, CBAM first derives one-dimensional channel attention $M_{c}$ from F, and two-dimensional spatial attention $M_{s}$ is introduced after multiplying F and $M_{c}$. F is multiplied again to obtain the output feature map. The output feature map has the same dimensions as the input feature map F. The calculation equation is shown in Equations (2) and (3).

(2) $$F^{\prime }=M_{c}(F)⊗F$$

(3) $$F^{\prime \prime }=M_{s}(F^{\prime })⊗F^{\prime }$$

In order to gather spatial information and feature cues of different objects to obtain more accurate channel attention, the average pooling and the max pooling are simultaneously used to compress the spatial dimension of the input feature map. The pooling vector is fed into a multi-layer perceptron and a hidden layer. The feature vector output from MLP is added elementwise and activated by sigmoid to obtain channel attention $M_{c}$. The calculation respects Equation (4).

(4) $$M_{c}(F)=\sigma ((MLP(AvgPool(F))+MLP(MaxPool(F)))$$

Spatial attention contains location information and is the supplement of channel attention. The obtained channel attention $M_{c}$ applies average pooling and max pooling operations along the channel axis. The generated effective feature vectors obtain spatial attention $M_{s}$ after convolution operation and sigmoid activation. The computing method is shown in Equation (5).

(5) $$M_{s}(F)=\sigma f(f^{7\times 7}([AvgPool(F)));(MaxPool(F)]))=\sigma (f^{7\times 7}([F_{avg}^{s};F_{\max}^{s}]))$$

### 2.3. Swin Transformer

**Figure 2:**
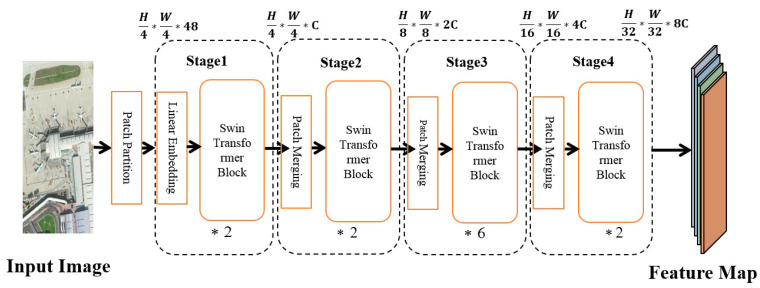
The architecture of the Swin Transformer.

The Swin Transformer [33] module consists of a multi-layer perceptron, a Window Multi-head Self-Attention (WMSA), a Shifted Window-based Multi-head Self Attention (SWMSA), and a Layer Normalization (LN). The workflow is shown as Figure 2.

**Figure 3:**
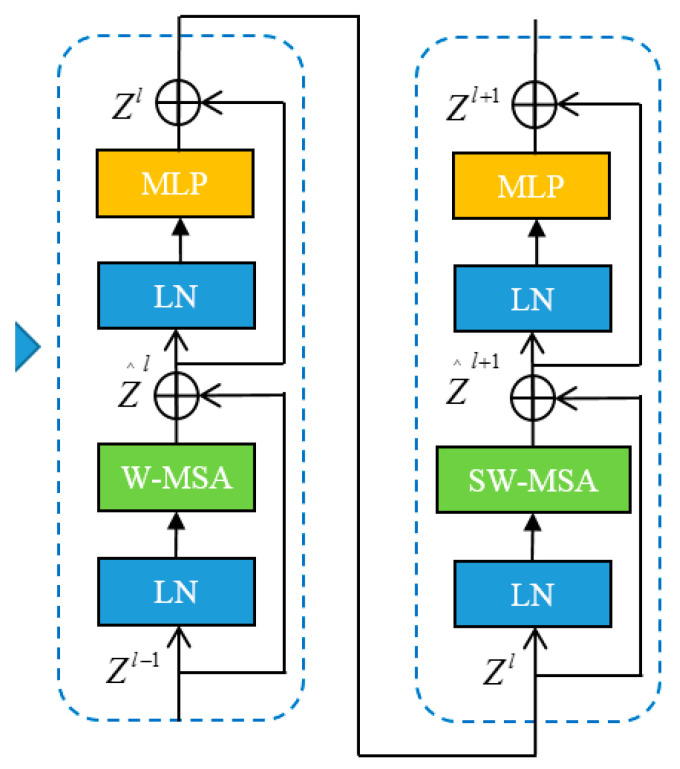
The architecture of the two blocks of the Swin Transformer.

The architecture of the two blocks of the Swin Transformer is shown in Figure 3.

First, the input image is normalized through the normalization layer. Then, the feature is learned through the W-MSA module, and the residual is calculated, which is then fed into the MLP through a layer of LN. Finally, the residual operation is performed again to obtain this layer’s output features. The architecture and workflow of SWMSA are similar to those of WMSA, and the difference is that SWMSA needs to perform a sliding window operation when calculating the feature part. In addition, WMSA and SWMSA are used in pairs, so the number of stacked Swin Transformer Blocks is even; the calculation expressions of each part of the Swin Transformer Backbone network are shown in Equations (6)–(9).

(6) $$Z^{{}^{\prime }l}=W-MSA(LN(Z^{l-1}))+Z^{l-1}$$

(7) $$Z^{l}=MLP(LN(Z^{{}^{\prime }l}))+Z^{{}^{\prime }l}$$

(8) $$Z^{{}^{\prime }l+1}=SW-MSA(LN(Z^{l}))+Z^{l}$$

(9) $$Z^{l+1}=MLP(LN(Z^{{}^{\prime }l+1}))+Z^{{}^{\prime }l+1}$$

where $Z^{{}^{\prime }l}$ and $Z^{l}$, respectively, represent the output features corresponding to module l, module (S)W-MSA, and module MLP. The Swin Transformer computes self-attention through local windows, which are arranged to evenly divide the image in a non-overlapping manner. Assuming that each window contains M*M small blocks, the computational complexities of an image based on a global-based MSA module and a h*w block are shown in Equations (10) and (11), respectively.

(10) $$\Omega (MSA)=4hwC^{2}+2{\left(hw\right)}^{2}C$$

(11) $$\Omega (W-MSA)=4hwC^{2}+2M^{2}hwC$$

From Equations (10) and (11), it can be clearly seen that, compared with the MSA module, the W-MSA design can save a considerable amount of computation. 

## 3. The Proposed Method

**Figure 4:**
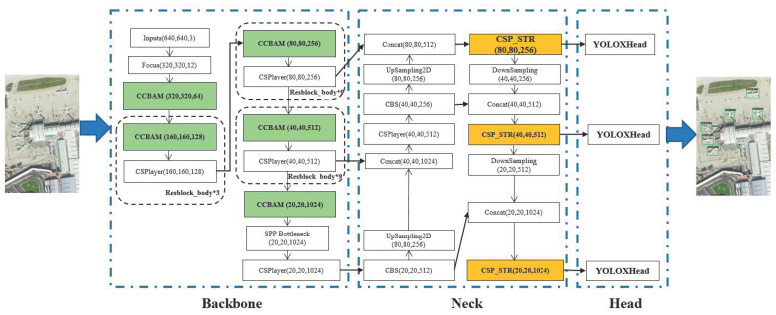
The architecture of the improved YOLOX.

In this section, we introduce the proposed Multiple Attention Mechanism Enhanced YOLOX (MAME-YOLOX) algorithm. The proposed MAME-YOLOX mainly contains three contributions. Firstly, we introduce the CBAM attention mechanism into the backbone of the YOLOX, so that the detection network can focus on the saliency information. Secondly, based on the idea of the self-attention mechanism, the Swin Trans-former is incorporated into the YOLOX’s neck module. Thirdly, instead of GIOU loss, the CIoUloss is adopted to measure the bounding box regression loss, which can prevent the GIoU from degenerating into IoU. The architecture of the improved YOLOX is shown in Figure 4.

### 3.1. CBAM Enhanced Feature Extraction

**Figure 5:**
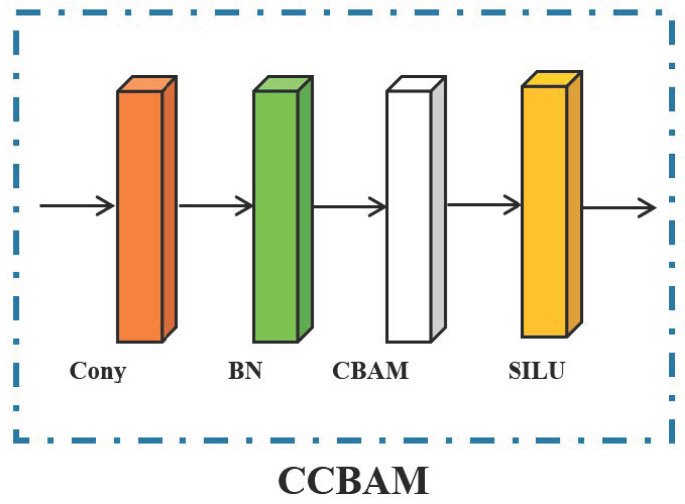
The architecture of the CCBAM.

Due to the complex background of the remote sensing image itself, in the process of multiple convolution operations, the iterations of the background will generate a large amount of redundant information, which conceals the valuable information of the image, resulting in a decrease in average precision. Therefore, this paper introduces the CBAM (Convolution Block Attention Module) attention mechanism into the convolutional block of the backbone of YOLOX, which can reduce the redundant information of the fully connected layer and enable the detection network to be more concerned about the locations that need attention. Moreover, it dramatically improves the accuracy of object detection and recognition, especially the precision of small objects. The architecture of the CCBAM is shown in Figure 5.

### 3.2. Self-Attention Enhanced Feature Representation

**Figure 6:**
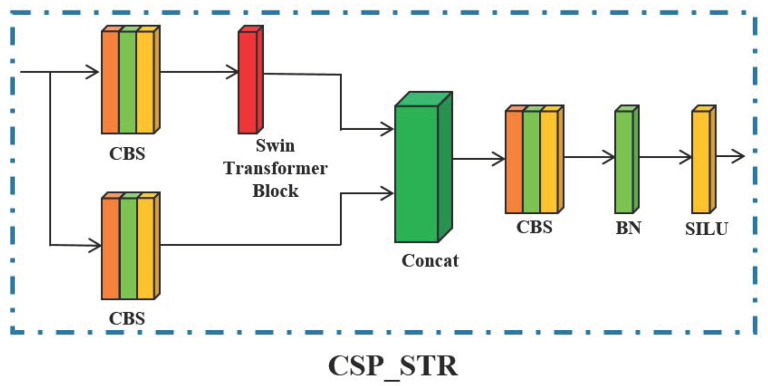
The architecture of the CSP_STR module.

Due to the dense objects in remote sensing images, with the deepening of the net-work and multiple convolution operations, most of the feature information of small objects is lost in the high-level feature map, which is prone to missed and false detections. The Swin Transformer uses a multi-head self-attention module to enhance the semantic information and feature representation of small objects in remote sensing images, which can strengthen the local perception ability of the network and improve the detection accuracy of small-scale objects. Compared with the traditional Transformer, the amount of calculation is significantly minimal. Therefore, the Swin Transformer module idea is introduced into the feature fusion of YOLOX’s neck. The YOLOX’s neck is based on CSP architecture. On this basis, we introduced the Swin Transformer into the CSP, which is called CSP_STR. The architecture of the CSP_STR module after introducing the Swin Transformer into the neck feature fusion network of YOLOX is shown as Figure 6.

The CSP_STR controls the computing area in each window by dividing local windows to realize cross-window information interaction, reduces computational complexity and network computation, and enhances the semantic information and feature representation of small objects.

### 3.3. Loss Function

The CIOU loss is used as the regression loss function of the bounding box, which solves the problem of GIOU degenerating into IOU. The definition of CIOU loss is shown in Equations (12)–(14).

(12) $$CIOU=1-IOU+\frac{p^{2}(b_{1}+b_{2})}{c^{2}}+\alpha v$$

(13) $$\alpha =\frac{v}{(1-IOU)+v}$$

(14) $$v=\frac{4}{\pi^{2}}{\left(\arctan\frac{w^{b_{1}}}{h^{b_{1}}}-\arctan\frac{w^{b_{2}}}{h^{b2}}\right)}^{2}$$

where $b_{1}$ is the center point of the prediction box, $b_{2}$ is the center point of the ground truth, and c is the diagonal distance of the smallest box covering the two frames. CIOU loss considers the Euclidean distance between the prediction and the ground truth. When the prediction is inside the ground truth, the position of the prediction is different, and the position of its center is also different, and CIOU loss can be calculated. $w^{b_{1}}$, $h^{b_{1}}$, $w^{b_{2}}$, and $h^{b2}$ are the width and height of the prediction box and the ground truth box, respectively. By introducing v, the aspect ratio between the predicted box and the object box is also taken into account when the centers of the two boxes coincide, which makes the positioning frame more accurate and improves detection accuracy. In addition, the classification loss of the proposed method is cross entropy (CE).

## 4. Experimental Results and Analysis

### 4.1. Datasets

**Figure 7:**
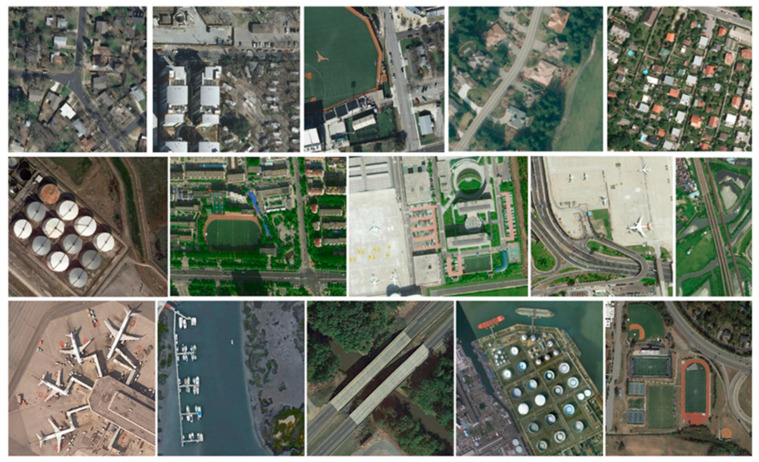
Sample images for three datasets. The first row’s dataset is AIBD, the second row’s dataset is HRRSD, and the third row’s dataset is DIOR.

Three publicly available object-detection datasets of remote sensing images, namely DIOR [34], HRRSD [35], and AIBD [36], are used to evaluate the proposed methods in the experiments. Some examples of DIOR, HRRSD, and AIBD are shown as Figure 7.

The AIBD dataset is dedicated to self-annotation for building detection tasks. AIBD was proposed for the first time in a category containing a single object: buildings. The sample size is 500 × 500, and there are 11,571 samples in total, the same number as the annotation files. Based on the COCO dataset standard, buildings are classified into large, medium, and small. The numbers of large, medium, and small instances are 16,824, 121,515, and 51,977, respectively. They are distinguished from each other by different colors with vastly different backgrounds. The number of pixels of buildings ranges from tens to hundreds of thousands. The geometric shapes of these building instances are diverse, including irregular shapes, such as U-shapes, T-shapes, and L-shapes. The raw data of AIBD are from the Inria aerial image data, accessible on 1 August 2020, mainly for se-mantic segmentation; training sets and test sets were selected from five cities. For each city, about 81 square kilometers of regions and 36 image blocks were selected. The training and test sets contained 180 image patches covering 405 km^2^. The resolution of each image block was 5000 × 5000, and the geographic resolution was 0.3 m. 

The HRRSD dataset was released by the University of Chinese Academy of Sciences in 2019. The dataset contains 21,761 images sampled from Google Earth and Baidu Maps, and the spatial resolution of these images ranges from 0.15 to 1.2 m. The dataset contains 55,740 instances covering 13 different object categories. These categories are airplanes, baseball fields, intersections, surface fields, basketball courts, bridges, ships, storage tanks, ports, parking lots, tennis courts, T-junctions, and vehicles. The biggest highlight of this dataset is that it has balanced samples under the category that cannot be used, and each category has nearly 4000 samples. In addition, the sample count of the training subset of this dataset is 5401, and the sample counts of the validation and test subsets are 5417 and 10943, respectively. The “training values” subset is the combination of the training and validation subsets. 

The DIOR dataset is a large-scale benchmark dataset mainly used for object detection in remote sensing images. Northwestern Polytechnical University in China released DIOR through sampling on Google Earth. The dataset contains 23,463 images, 20 object classes, and 192,472 instances. The 20 object categories include airplanes, baseball fields, basketball courts, airports, bridges, chimneys, highway service areas, dams, highway tollbooths, ground track fields, seaports, golf courses, flyovers, stadiums, storage tanks, ships, tennis courts, vehicles, railway stations, and windmills. The dataset image size is 800 × 800, with a spatial resolution from 0.5 to 30 m. This dataset has four salient features: (1) it contains a large number of object instances and images, (2) a variety of different object scales, and (3) different weather, imaging conditions, seasons, etc., and (4) it has high intra-class diversity and inter-class similarity.

In this paper, the percentage of the training set, validation set, and test set of the DIOR dataset is 0.25, 0.25, and 0.5, respectively. The training set and verification set in the other two datasets, HRRSD and AIBD, are jointly used for model training.

### 4.2. Evaluation Metrics

In this paper, the mAP (mean average precision) and mAP_50 are selected as the main indicators of the experimental results. The evaluation metrics used are from the standard COCO metric set. We also choose mAP_75, mAP_s, mAP_m, and mAP_l as the evaluation indicators of the experimental results. mAP_50 and mAP_75 indicate the accuracy, with a threshold value of 0.5 and 0.75, respectively. mAP_s demonstrates that the average accuracy of the object is small (smaller than 322); mAP_m represents that the average accuracy is at a medium level (between 322 and 962); and mAP_l expresses that the average accuracy is large (bigger than 962). mAP is an indicator for measuring recognition accuracy in object detection, and is the average of AP for multiple categories. mAP is defined in Equation (15).

(15) $$mAP=\frac{\sum_{q=1}^{Q}AveP(q)}{Q}$$

where Q represents the category set of object detection and AveP(q) is the average accuracy rate of the object under the calculation category. Precision-recall (PR) is calculated as shown in Equations (16) and (17).

(16) $$Precision=\frac{TP}{TP+FP}$$

(17) $$\mathrm{Recall}=\frac{TP}{TP+FN}$$

where true positive (TP) represents the number of positive samples that are correctly predicted as positive. True negative (TN) represents the number of negative samples that are correctly predicted as negative. False positive (FP) represents the number of negative samples that are incorrectly predicted as positive. False negative (FN) represents the number of positive samples that are incorrectly predicted as negative.

In addition, the TP, TN, FP, and FN of object detection are derived by the IOU (intersection over union). The IOU measures the overlap rate between two regions within the object detection range, as shown in Equation (18).

(18) $$IOU=\frac{area(B_{p}\cap B_{gt})}{area(B_{p}\cup B_{gt})}$$

where IOU represents the overlapping area between the predicted bounding box $B_{p}$ and the true bounding box $B_{gt}$ divided by the union area of the two. The predicted bounding boxes are classified as true or false by comparing the IOU with a given threshold *T*. If IOU ≥ *T*, it is considered true. If the contrary, the detection is considered false.

### 4.3. Experimental Setups

The competitive algorithms include Faster R-CNN [37], RetinaNet [38], SSD512 [39], YOLOv3 [16], and YOLOX [18]. Among them, Faster R-CNN is a typical representative of two-stage methods, while RetinaNet, SSD512, YOLOv3, and YOLOX are representative of single-stage methods. In the three datasets for which we visualized test results, the true cases (TPs), false positive cases (FPs), and false negative cases (FNs) are represented by green boxes, red boxes, and yellow boxes, respectively. The parameter settings of the competitive algorithms are summarized in Table 1.

The methods used in the comparison experiment are based on the MMdetection platform (https://github.com/open-mmlab/mmdetection, accessed on 21 October 2022). The host computer for model training consists of four graphical processors of Nvidia GeForce RTX 2080.

### 4.4. Experimental Analysis

**Figure 8:**
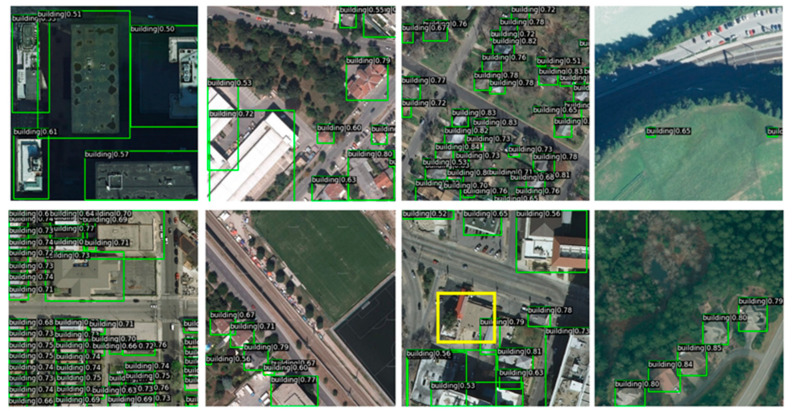
The AIBD dataset’s visualized test results using different algorithms.

Firstly, the improved algorithm proposed in this paper is compared with the object detection algorithm mentioned above in the AIBD dataset. The visualized test results are shown in Figure 8.

In Figure 8, although AIBD is a building object detection dataset containing a single category, the intra-category differences are very large. It can be seen that the apparent characteristics of buildings greatly vary, whether for common rectangular buildings or for buildings with irregular shapes. However, the overall test result of MAME-YOLOX is satisfactory. The yellow rectangular box is the object of the undetected building, which is a false negative (FNs). Because the color of the building is very similar to the surrounding roads, the edge features are not clear.

The quantitative comparison experiment results of the AIBD dataset are shown in Table 2 and Table 3. Table 2 presents algorithm, backbone, mAP, and mAP_50 values. Table 3 presents the mAP_75, mAP_s, mAP_m, and mAP_l values.

From Table 2, we can clearly see MAME-YOLOX has the best effect on the two main indicators of mAP and mAP_50, which are 0.479 and 0.848, respectively. Moreover, from Table 2, we can see for different object sizes; MAME-YOLOX’s mAP_m indicator is optimal. The second-best results of mAP and mAP_50 were obtained by YOLOX, which are 0.467 and 0.832, respectively. Compared with the YOLOX algorithm, MAME-YOLOX’s overall mAP value increased by 1.8%, while the mAP_50 value increased by 1.7%.

**Figure 9:**
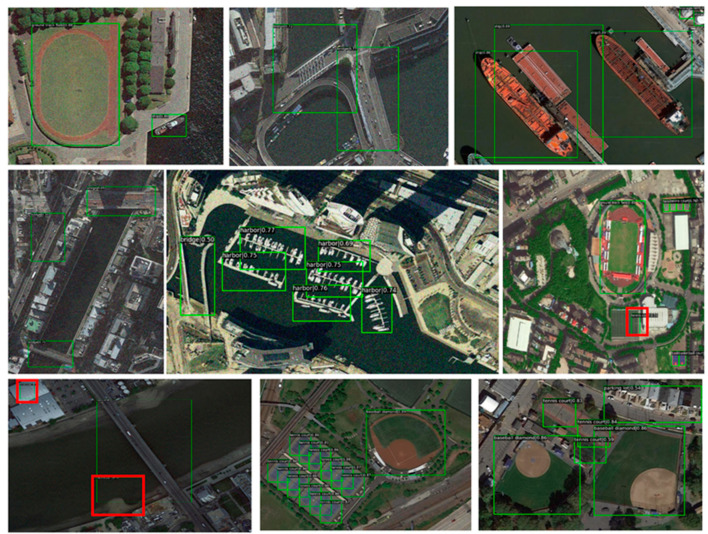
The HRRSD dataset’s visualized test results using different algorithms.

Secondly, the improved algorithm proposed in this paper is compared with the object detection algorithm mentioned above in the HRRSD dataset, which has 13 object categories. The visualized test results are shown in Figure 9.

In Figure 9, we can see most of the objects of the HRRSD dataset have obvious features, such as apparent texture and color, and the size of the object is large. However, information-rich high-score images also have the problems of small differences between categories, large differences within categories, and it being difficult to unify semantics. Therefore, the red rectangular boxes in Figure 9 are all examples of false negative detections. Among them, the first one is that the highway is wrongly detected as a bridge. From the visual observation, its foreground and background information is very similar to the bridge. In the second red rectangle, the road turntable is mistakenly detected as a storage tank. If the context information of the object image is fully considered, the object should not be mistakenly detected. The quantitative comparison experiment results on HRRSD datasets are shown in Table 4 and Table 5. Table 4 presents the algorithm, backbone, mAP, and mAP_50 value. Table 5 presents the mAP_75, mAP_s, mAP_m, and mAP_l values.

From Table 4, it can be seen that Faster R-CNN obtained the best mAP and mAP_50 indicator results, at 0.561 and 0.888, respectively. MAME-YOLOX ranks second, and the results are 0.549 and 0.862, respectively. However, our algorithm processing speed is better than that of Faster R-CNN. Compared with the YOLOX algorithm, MAME-YOLOX’s overall mAP value increased by 1.4%, while the mAP_50 value increased by 1.6%. 

**Figure 10:**
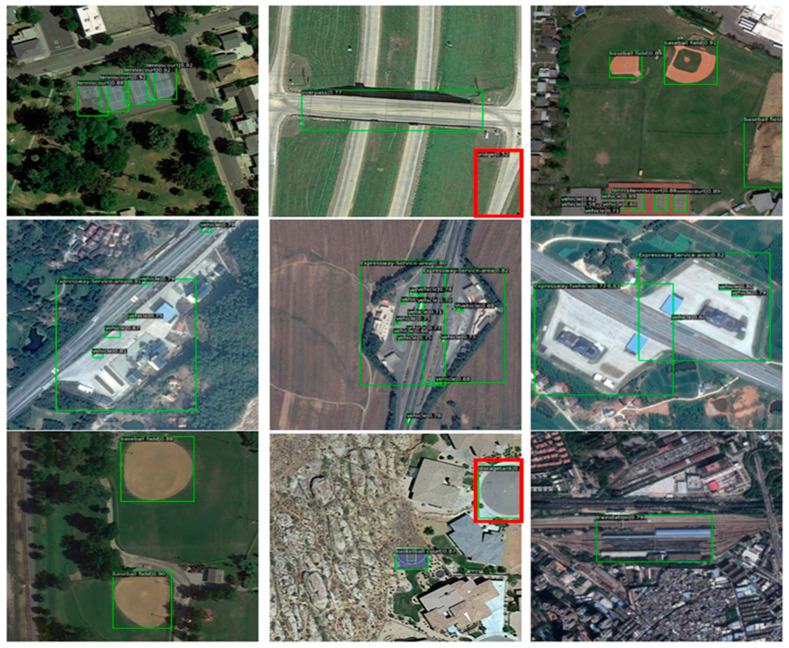
The DIOR dataset’s visualized test results using different algorithms.

Thirdly, the improved algorithm proposed in this paper is compared with the object detection algorithm mentioned above in the DIOR dataset. The visualized test results are shown in Figure 10.

The DIOR dataset contains 20 object categories, and in Figure 10 we can see that when the object instance has the characteristics of a small size and a dense appearance, such as vehicles, ports may be detected by mistake. However, objects with relatively fixed appearance features, such as aircrafts and tanks, are rarely detected by mistake or omitted. The quantitative comparison experiment results of the DIOR dataset are shown in Table 6 and Table 7. Table 6 provides the algorithm, backbone, mAP, and mAP_50 value. Table 7 presents the mAP_75, mAP_s, mAP_m, and mAP_l values.

From Table 6, we can see MAME-YOLOX obtains the optimal mAP indicator, 0.501, and the optimal mAP_50 indicator, 0.772, respectively. Second place is obtained by the SSD512 algorithm, where the mAP result is 0.497 and mAP_50 is 0.759. Compared with the YOLOX algorithm, MAME-YOLOX’s overall mAP value increased by 3.2%, while the mAP_50 value increased by 3.4%.

The ablation experiments on the AIBD, HRRSD, and DIOR datasets are shown in Table 8, Table 9 and Table 10, respectively.

The experimental results demonstrate that the architecture of the proposed method is effective and achieves better results. For example, the mAP and mAP_50 of the MAME-YOLOX are 0.273 and 0.472, respectively, being better than those of the YOLOX, YOLOX + SwinTrans., and YOLOX + CBAM. The SwinTrans. module and CBAM module are all beneficial to improve the performance; however, the MAME-YOLOX achieves the best results.

From the qualitative visualization examples of the three datasets, the proposed framework in this paper fits more closely with the remote sensing object to be detected. At the same time, this method can detect some small remote sensing objects that are not easy to find, which reduces the missed detection rate of small objects to a certain extent, and thus improves the detection accuracy of remote sensing objects. MAME-YOLOX is a very promising method, which has a strong ability for object detection in remote sensing images. It is not only applicable to datasets of multiple object categories, but also applicable to datasets of single object categories.

## 5. Conclusions

Aiming at detection robustness for tiny objects against complex backgrounds, in this paper, we proposed a Multiple Attention Mechanism Enhanced YOLOX (MAME-YOLOX) algorithm. Firstly, the CBAM attention mechanism was introduced into the convolution block of YOLOX’s backbone to reduce the redundant information. This mechanism could improve the detection accuracy. Secondly, the idea of the Swin Transformer was integrated into the feature fusion of YOLOX’s neck to enhance the global perception for small objects. This design produced a better fitting effect on small objects. Finally, we used CIoU loss to replace the GIOU loss as the regression loss of the bounding box, enhancing the accuracy of object detection. The experimental results showed that the mAP of the MAME-YOLOX improved the original YOLOX by 1.8, 1.4, and 3.2% on the three datasets, AIBD, HRRSD, and DIOR, respectively. This demonstrated the effectiveness of MAME-YOLOX for remote sensing object detection. 

In the future, we will explore lightweight feature extraction networks and simplify the network architecture.

## Figures and Tables

**Table 1:** The parameter settings of the competitive algorithms.

Algorithm	LR	Decay	BS	Classification Loss	Bounding Box Loss	Optimizer
Faster R-CNN	0.02	0.0001	16	Cross Entropy	L1loss	SGD
RetinaNet	0.01	0.0001	16	Focal Loss	L1loss	SGD
SSD512	0.002	0.0005	16	Cross Entropy	SmoothL1	SGD
YOLOv3	0.002	0.0002	16	Cross Entropy	MSELoss	SGD
YOLOX	0.01	0.0005	16	Cross Entropy	GIOU loss	SGD
MAME-YOLOX	0.02	0.0001	16	Cross Entropy	CIOU loss	SGD

**Table 2:** Comparative experimental results of different algorithms on the AIBD dataset.

Algorithm	Backbone	Inference Time per Image (Secs.)	mAP	mAP_50	mAP_75
Faster R-CNN	resnet50	0.052	0.465	0.813	0.502
RetinaNet	resnet50	0.037	0.439	0.798	0.423
SSD512	resnet50	0.031	0.439	0.805	0.424
YOLOv3	Darknet53	0.028	0.429	0.808	0.402
YOLOX	CSPDarknet	0.022	0.461	0.831	0.497
MAME-YOLOX	CSPDarknet	0.023	0.479	0.848	0.485

**Table 3:** Comparative experimental results of different algorithms on the AIBD dataset.

Algorithm	Backbone	mAP_s	mAP_m	mAP_l
Faster R-CNN	resnet50	0.356	0.501	0.546
RetinaNet	resnet50	0.338	0.472	0.483
SSD512	resnet50	0.341	0.478	0.494
YOLOv3	Darknet53	0.311	0.476	0.396
YOLOX	CSPDarknet	0.367	0.506	0.502
MAME-YOLOX	CSPDarknet	0.359	0.516	0.506

**Table 4:** Comparative experimental results of different algorithms on HRRSD dataset.

Algorithm	Backbone	Inference Time per Image (Secs.)	mAP	mAP_50	mAP_75
Faster R-CNN	resnet50	0.061	0.561	0.888	0.704
RetinaNet	resnet50	0.043	0.514	0.810	0.537
SSD512	resnet50	0.039	0.521	0.797	0.567
YOLOv3	Darknet53	0.035	0.505	0.827	0.518
YOLOX	CSPDarknet	0.024	0.535	0.846	0.524
MAME-YOLOX	CSPDarknet	0.027	0.549	0.862	0.607

**Table 5:** Comparative experimental results of different algorithms on HRRSD dataset.

Algorithm	Backbone	mAP_s	mAP_m	mAP_l
Faster R-CNN	resnet50	0.231	0.506	0.581
RetinaNet	resnet50	0.128	0.460	0.513
SSD512	resnet50	0.096	0.416	0.501
YOLOv3	Darknet53	0.081	0.391	0.502
YOLOX	CSPDarknet	0.270	0.443	0.509
MAME-YOLOX	CSPDarknet	0.273	0.472	0.533

**Table 6:** Comparative experimental results of different algorithms on the DIOR dataset.

Algorithm	Backbone	Inference Time per Image (Secs.)	mAP	mAP_50	mAP_75
Faster R-CNN	resnet50	0.059	0.415	0.678	0.448
RetinaNet	resnet50	0.041	0.313	0.539	0.336
SSD512	resnet50	0.038	0.497	0.759	0.536
YOLOv3	Darknet53	0.032	0.334	0.651	0.288
YOLOX	CSPDarknet	0.023	0.469	0.738	0.522
MAME-YOLOX	CSPDarknet	0.025	0.501	0.772	0.547

**Table 7:** Comparative experimental results of different algorithms on the DIOR dataset.

Algorithm	Backbone	mAP_s	mAP_m	mAP_l
Faster R-CNN	resnet50	0.073	0.254	0.529
RetinaNet	resnet50	0.041	0.199	0.411
SSD512	resnet50	0.078	0.311	0.606
YOLOv3	Darknet53	0.061	0.207	0.392
YOLOX	CSPDarknet	0.084	0.310	0.499
MAME-YOLOX	CSPDarknet	0.101	0.355	0.602

**Table 8:** The ablation experiments on the AIBD dataset.

Algorithm	mAP	mAP_50	mAP_75
YOLOX	0.461	0.831	0.497
YOLOX + SwinTrans.	0.463	0.836	0.492
YOLOX + CBAM	0.465	0.834	0.488
MAME-YOLOX	0.479	0.848	0.485

**Table 9:** The ablation experiments on the HRRSD dataset.

Algorithm	mAP	mAP_50	mAP_75
YOLOX	0.270	0.443	0.509
YOLOX + SwinTrans.	0.272	0.452	0.524
YOLOX + CBAM	0.269	0.468	0.517
MAME-YOLOX	0.273	0.472	0.533

**Table 10:** The ablation experiments on the DIOR dataset.

Algorithm	mAP	mAP_50	mAP_75
YOLOX	0.084	0.310	0.499
YOLOX + SwinTrans.	0.096	0.345	0.561
YOLOX + CBAM	0.088	0.337	0.589
MAME-YOLOX	0.101	0.355	0.602

## Data Availability

Not applicable.
